# Epigenetic Instability due to Defective Replication of Structured DNA

**DOI:** 10.1016/j.molcel.2010.11.009

**Published:** 2010-12-10

**Authors:** Peter Sarkies, Charlie Reams, Laura J. Simpson, Julian E. Sale

**Affiliations:** 1 Medical Research Council Laboratory of Molecular Biology, Hills Road, Cambridge CB2 0QH, UK; 2 University of Cambridge Computer Laboratory, William Gates Building, 15, J.J. Thomson Avenue, Cambridge CB3 0FD, UK

## Abstract

The accurate propagation of histone marks during chromosomal replication is proposed to rely on the tight coupling of replication with the recycling of parental histones to the daughter strands. Here, we show in the avian cell line DT40 that REV1, a key regulator of DNA translesion synthesis at the replication fork, is required for the maintenance of repressive chromatin marks and gene silencing in the vicinity of DNA capable of forming G-quadruplex (G4) structures. We demonstrate a previously unappreciated requirement for REV1 in replication of G4 forming sequences and show that transplanting a G4 forming sequence into a silent locus leads to its derepression in REV1-deficient cells. Together, our observations support a model in which failure to maintain processive DNA replication at G4 DNA in REV1-deficient cells leads to uncoupling of DNA synthesis from histone recycling, resulting in localized loss of repressive chromatin through biased incorporation of newly synthesized histones.

## Graphical abstract

**Graphical Abstract:**

**Figure:**
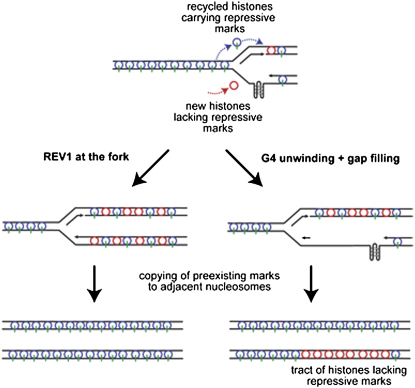

**Highlights:**

► REV1 facilitates replication of G4 DNA blocking the leading strand ► *rev1* cells exhibit impaired recycling of repressive histone modifications at G4 DNA ► Transcriptional derepression across the genome in *rev1*cells correlates with G4 DNA ► Inserting a G4 DNA sequence leads to derepression of a silent locus in *rev1* cells

## Introduction

Multicellular organisms must maintain gene expression states, and therefore cell identity, epigenetically through cell division (Corpet and Almouzni, 2009). It is proposed that this is achieved through posttranslational modification of the histone proteins around which the DNA is wrapped in chromatin. During replication, histones are displaced by the replicative helicase and then randomly distributed to the nascent daughter DNA strands in a process coordinated by histone chaperones, notably Asf1 and Caf1 (reviewed in De Koning et al., 2007). To avoid a reduction in nucleosome density, recycled histones are combined with newly synthesized histones. The modifications on the parental histones can then be copied to the new histones (Bannister et al., 2001; Hansen et al., 2008; Lachner et al., 2001; Margueron and Reinberg, 2010). In order for this process to be viable as a mechanism of maintaining gene expression states, it is essential that there be coordination between histone eviction by the replicative helicase and the synthesis of new DNA. Without this coordination, parental histones will not be deposited near to their original locations, resulting in loss of the epigenetic information carried by their posttranslational modifications.

DNA replication is susceptible to interruptions caused by, for example, DNA damage. In turn, replication arrest interrupts histone recycling (Jasencakova et al., 2010). The Y family DNA polymerase REV1 plays an important role in vertebrates in maintaining replication fork progression on damaged DNA templates (Edmunds et al., 2008; Jansen et al., 2009), a role it fulfils by coordinating other specialized polymerases that are able to bypass DNA damage directly in a process known as translesion synthesis (Guo et al., 2003; Ross et al., 2005). In REV1-deficient cells, this results in damage bypass taking place predominantly in postreplicative gaps (Edmunds et al., 2008), which form when replication restarts downstream of a block. Importantly, the DNA synthesis associated with the filling of these gaps, which have been estimated to be between about 400 and 3000 bp in length and visualized to persist up to 20 kb behind the fork (Lehmann, 1972; Lopes et al., 2006), will be uncoupled from bulk DNA replication and therefore from the replicative helicase and histone recycling. Indeed, in budding yeast, gap filling can be deferred to G2 (Daigaku et al., 2010; Karras and Jentsch, 2010). Thus, chromatinization of these regions will be likely to exhibit a bias toward the deposition of newly synthesized histones and therefore result in the formation of a tract of nucleosomes lacking key epigenetic marks present in the parental strands.

We postulated that REV1-deficient cells would be more liable to loss of coordination between the replicative helicase and DNA synthesis, and that this might lead to loss of chromatin modifications through the preferential incorporation of new, unmodified histones during gap filling. To test this hypothesis, we have taken advantage of the chicken β-globin locus, in which the histone modifications associated with developmentally regulated expression have been extensively studied (reviewed in Felsenfeld, 1993; Felsenfeld et al., 2004) and the genetic tractability of the chicken cell line DT40 (Buerstedde and Takeda, 1991). We therefore set out to ask initially whether loss of REV1 had any impact on the ability of DT40 to maintain repression of the β-globin locus.

## Results

### Loss of H3K9 Dimethylation in the β-Globin Locus of *rev1* Cells Is Associated with an Increase in Marks of New Histone Deposition

**Figure 1:**
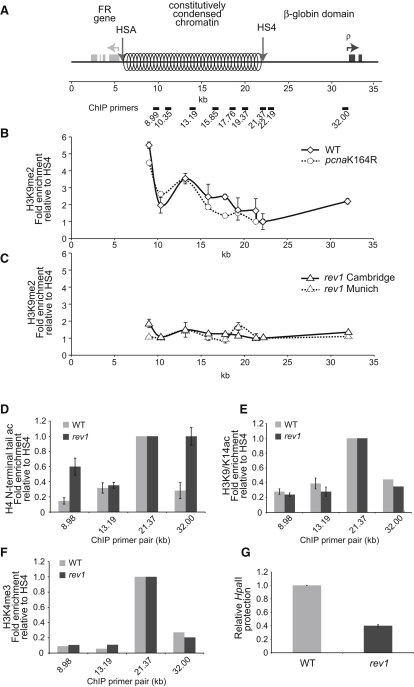
Epigenetic Dysfunction in the β-Globin Locus of *rev1* DT40 (A) Map of the region of the chicken β-globin locus studied in this paper (Litt et al., 2001b) between the folate receptor (FR) gene and first of the β-globin genes, ρ. HSA and HS4 are DNase hypersensitive sites that correspond with chromatin domain insulator sequences (Felsenfeld et al., 2004). The distance markers and location of the ChIP primers are indicated below the diagram (see also Table S2). (B) H3K9 dimethylation (H3K9me2) in the WT (solid line) and *pcna*K164R (dashed line). In all cases, the specific ChIP signal was normalized to total H3 then to the signal at HS4 (21.37). Error bars represent the standard error of the mean. (C) H3K9 dimethylation (H3K9me2) in *rev1* cells derived in our laboratory (solid line, “*rev1* Cambridge”) (Simpson and Sale, 2003) and independently the laboratory of Jean-Marie Buerstedde (dashed line, “*rev1* Munich”) (Arakawa et al., 2006). (D) Increased levels of acetylation of the N terminus of H4 in *rev1* cells. (E) Acetylation of H3 at K9 and K14 in WT and *rev1* cells. (F) Trimethylation of H3 at K4 in WT and *rev1* cells. (G) Loss of DNA methylation at the ρ-globin promoter in *rev1* cells. Loss of DNA methylation renders the ρ-globin promoter sensitive to restriction by HpaII. Amplification of the ρ-globin promoter by qPCR after HpaII restriction allowed the fraction of DNA remaining uncleaved, and therefore methylated, to be determined. Amplification was normalized to BamHI digested genomic DNA, then further normalized to set the WT level at 1. The amplified region does not contain any BamHI sites. Error bars represent the standard deviation. See also Figure S1.

To test the hypothesis that *rev1* cells might lose epigenetic information, we used chromatin immunoprecipitation (ChIP) to examine the histone modifications at the β-globin locus (Figure 1A), which has been previously shown to be silent in nonerythroid cells (Litt et al., 2001a), and specifically in DT40 (Litt et al., 2001b). Consistent with this, we found an enrichment of the repressive H3K9me2 modification in wild-type (WT) DT40 cells across the constitutively condensed chromatin domain and at the promoter of the nearby ρ-globin gene (Figure 1B). Comparable enrichment was evident in a cell line, *pcna*K164R, in which PCNA cannot be ubiquitinated and which is defective in postreplicative gap filling (Arakawa et al., 2006; Edmunds et al., 2008) (Figure 1B). In contrast, we observed a greatly reduced enrichment of H3K9me2 in two independently generated *rev1* lines (Arakawa et al., 2006; Simpson and Sale, 2003) (Figure 1C). Associated with loss of H3K9me2, there was enrichment in acetylation of the H4 N-terminal tail in *rev1* compared to WT DT40 (Figure 1D). As the increase in H4 N-terminal acetylation was not associated with enrichment of other marks of canonical transcriptional activation, H3K9/14ac and H3K4me3, previously observed at this locus (Litt et al., 2001a; Litt et al., 2001b) (Figures 1E and 1F), it is consistent with enrichment of newly synthesized histones in *rev1* cells relative to the WT (Lande-Diner et al., 2009; Sobel et al., 1995). Interestingly, we also observed an increase in H3K56 acetylation (Figure S1 available online), which is a robust marker of newly synthesized H3 in yeast (Li et al., 2008) but whose significance in vertebrates remains a subject of debate. Despite not observing histone marks associated with transcriptional activation, we did observe significant loss of DNA methylation at the ρ-globin promoter in *rev1* cells (Figure 1G).

### Derepression of the ρ-Globin Locus in REV1-Deficient Cells

**Figure 2:**
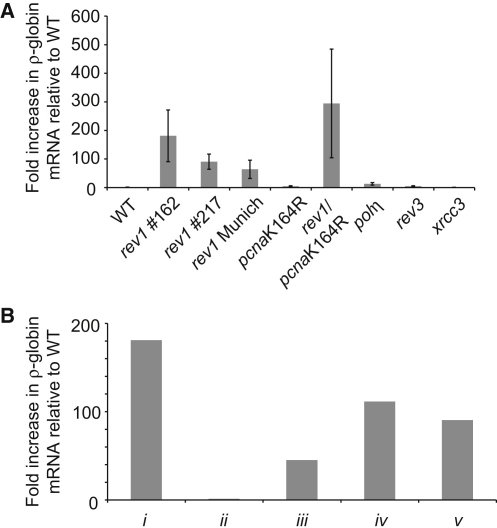
Derepression of ρ-Globin Expression in *rev1* Cells (A) Derepression of ρ-globin expression in *rev1* cells. Comparison of ρ-globin expression in different DT40 mutants. Expression, monitored by qRT-PCR with primers RhoExpF and R (Table S2), is given as the fold increase over the WT level, which is set at 1. *rev1* #162 and #217 are two independent *rev1* clones derived in our lab (“*rev1* Cambridge”). Error bars show the range. (B) Effect of complementation with human REV1 on ρ-globin derepression. Increase in expression of ρ-globin in *rev1* cells, and *rev1* cells complemented with hREV1, relative to WT: *i*, *rev1* cells cultured for >3 months; *ii*, *rev1* complemented with hREV1 at 4 weeks, which is as soon as practically possible, and then cultured for >3 months; *iii*, *rev1* cells from *i*, with established derepression of ρ-globin, complemented with hREV1 and cultured for 3 weeks; *iv*, as *iii*, but cultured for 3.5 weeks; and *v*, as *iii*, but cultured for 5 weeks. Five weeks in culture corresponds conservatively with 70 cell divisions.

Enrichment of H3K9me2 across the promoter of the ρ-globin gene is associated with its silencing (Litt et al., 2001a). Therefore, we predicted that loss of this modification in *rev1* cells would lead to increased expression of the gene. Quantitative PCR revealed an approximately 100-fold increase in the expression of the ρ-globin gene in *rev1* cells relative to WT cells (Figure 2A). Consistent with the enrichment of H3K9me2, no increase in ρ-globin expression was seen in the *pcna*K164R line. Moreover, a mutant lacking XRCC3, defective in homologous recombination, also showed no increase in ρ-globin expression. Interestingly, mutants defective in the translesion polymerases Polη and Polζ (REV3) showed only small increases in ρ-globin expression, which correlates with their mild phenotypes when assessing the progression of replication forks on damaged DNA templates (Edmunds et al., 2008; Jansen et al., 2009). Reintroduction of a human REV1 complementary DNA (cDNA), which we have previously shown to complement all phenotypes of the chicken *rev1* line (Edmunds et al., 2008; Ross et al., 2005), was unable to reverse the ρ-globin derepression once established over the course of 5 weeks in culture (conservatively 70 cell divisions), suggesting that the kinetics of restoration of repressive marks is, at best, slow (Figure 2B). However, a *rev1* line in which hREV1 had been reintroduced at an early stage but then cultured for several months did not exhibit ρ-globin derepression.

### Spontaneous DNA Damage Is Unlikely to Explain the Loss of Repressive Histone Marks in REV1-Deficient Cells

**Figure 3:**
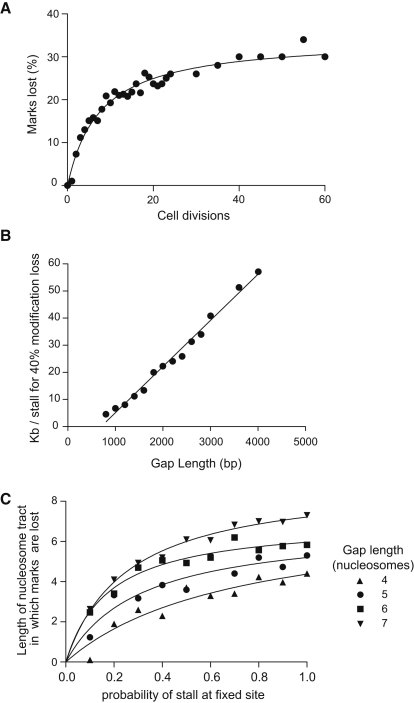
Computational Simulation of Loss of Histone Modifications in Response to Formation of Postreplicative Gaps (A) Percentage of marks lost as a function of time with a fixed stall probability. The graph shows a representative time course for histone modification loss. For this simulation, the postreplicative gap length was set at 1 kb, the probability of two place copy at 0.25 and the probability of fork stalling at 0.025 per nucleosome. This corresponds to one stall every 8 kb. Each data point represents an average of 30 simulations. (B) Postreplicative gap length necessary to produce 40% loss of histone marks in 30 generations. A lower estimate for spontaneous stalling intervals of 60–100kb would clearly place the necessary postreplicative gap length much higher than any current in vivo estimate (see the main text). The x axis scale assumes an internucleosome distance of 200 bp. (C) Length of nucleosome tract in which marks are lost as a function of the probability of stalling per replication cycle at a fixed point. Data is shown for a postreplicative gap length of four to seven nucleosomes, with the variance in length set to 0.

Taken together, these data are consistent with the hypothesis that gap-filling modes of DNA replication predominate in *rev1* DT40 cells, leading to the replacement of parental modified histones with newly synthesized histones devoid of repressive marks. However, the effect appears without the introduction of exogenous DNA damaging agents. We therefore asked whether spontaneous DNA damage could be sufficient to lead to this phenotype. To address this question theoretically, we developed a computer model simulating inheritance of histone modifications across cell division through copying of modifications from parental histones to newly synthesized histones (see the Experimental Procedures). We also simulated replication fork stalling, occurring with a defined probability per cell division, that leads to gap-filling DNA synthesis accompanied by a tract of newly synthesized histones with length equal to the length of the gap. We could vary the probability of replication fork stalling and the length of the gap to examine the effect these parameters might have on epigenetic stability. Our model showed that it was possible to obtain loss of histone modifications with random replication fork stalling using a gap length consistent with that observed in vivo, but only at a very high frequency of stalling (one stall every 10 kb) (Figure 3A). Even assuming stalling at the maximum level possible from estimates of the frequency of spontaneous damage, approximately one lesion per ∼60 kb, (Lindahl, 1996), the mean length of gap would have to be greater than 8 kb (approximately 40 nucleosomes) in length to achieve a 40% loss of histone modifications in our model (Figure 3B). This is considerably greater than the largest current estimate for the length of postreplicative gaps (Lopes et al., 2006).

We therefore considered the possibility that replication forks may stall more frequently at specific sites within the β-globin locus. Such sites exist widely in all genomes and are frequently found where the DNA sequence can form secondary structures (reviewed in Mirkin and Mirkin, 2007). Indeed, the chicken ρ-globin gene has been previously shown to contain a region in the second intron in which replication forks are slowed or blocked (Prioleau et al., 2003). We therefore simulated the effect of a fixed stall and found that, with a probability of stalling of 0.4 or above per cell division, a stable tract of lost histone modifications approximately equal to the length of the gap had developed after 30 cell divisions (Figure 3C).

### G Quadruplex Formation by the ρ-Globin Second Intron Sequence

**Figure 4:**
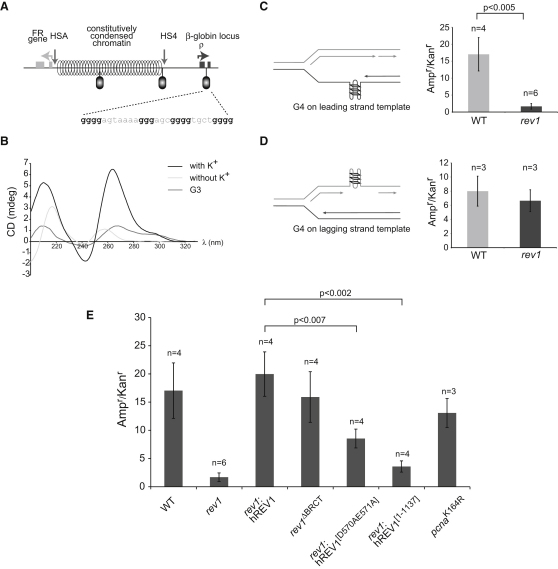
REV1 Is Required for Replication of G Quadruplex-Forming DNA on the Leading-Strand Template (A) Position of G4 DNAs (ovals) in the region of the β-globin locus studied in this work. The sequence of the ρ-globin G4 DNA is shown. (B) Circular dichroism spectroscopy of the ρ-globin G quadruplex forming sequence. Renaturation of the minimal 29 bp G4 oligonucleotide (GQCDG4) in the presence (black line) or absence (light gray line) of K^+^ ions. Renaturation of a truncated ρ-globin G4 sequence (GQCDG3) in the presence of K^+^ ions (mid gray line). (C and D)Replication efficiency, shown as the ratio of Amp^r^ to Kan^r^*E. coli* colonies, for the ρ-globin G4 DNA on the leading- (C) and lagging- (D) strand template of pQ (Szüts et al., 2008). Error bars represent standard error of the mean. p values were calculated using the unpaired t test (two-tailed). (E) Replication efficiency of the leading-strand template G4 in *rev1* mutants. Complementation is with full-length, catalytically inactive (D570AE571A) and C-terminally truncated (1-1137) human REV1. The BRCT mutant is an endogenous deletion of amino acids 69–116 of REV1 (Figure S2). The WT and *rev1* data from Figure 4C are shown again for comparison. Error bars represent standard error of the mean. See also Figures S2 and S3.

We therefore examined the sequence in the second intron of ρ-globin and noted, at the site identified by Prioleau et al. (2003), a sequence that corresponds to a consensus for a G quadruplex (G4) DNA (Figure 4A). Indeed, the region of the chicken β-globin locus we studied in this work contains three G4 sequences, the other two residing in the constitutively condensed region (Figure 4A). G4 DNA, of the general sequence G_3-5_-L_1-7_-G_3-5_-L_1-7_-G_3-5_-L_1-7_-G_3-5_ (where L can be any base), can form a variety of secondary structures at physiological salt concentrations, whose stability exceeds that of duplex DNA, both in vitro and in vivo (Lipps and Rhodes, 2009; Maizels, 2006). These structures are characterized by stacks of planar arrays of four Hoogsteen bonded dG bases coordinated by a monovalent metal ion (Sundquist and Klug, 1989; Williamson et al., 1989). In vitro, the 29bp G4 sequence from the ρ-globin intron forms a K^+^-dependent quadruplex structure as shown by circular dichroism spectroscopy, with diagnostic positive peaks at 210 nm and 265 nm (Kypr et al., 2009) (Figure 4B). This structure is dependent on four G-rich blocks in the oligonucleotide (Figure 4B).

### REV1 Is Required for Efficient Replication of G Quadruplex-Forming DNA on the Leading-Strand Template

Translesion synthesis has been previously implicated in the replication of G4 DNA (Bétous et al., 2009; Youds et al., 2006). We therefore asked whether REV1 assists in replication of this specific G4 DNA sequence. To do this, we took advantage of a replicating plasmid assay (Szüts et al., 2008), which can measure the efficiency of replication as a change in the number of ampicillin resistant colonies recovered normalized to a control kanamycin-resistant plasmid not containing the G4 sequence. We found a striking reduction in the efficiency of replication of the plasmid when the G4 sequence was placed on the leading-strand template, but not on the lagging-strand template (Figures 4C and 4D), implying a role for REV1 in the replication of this sequence. This observation is consistent with the presence of two strong origins mapped 3′ of the ρ-globin G4, which would place the G4 on the leading-strand template (Prioleau et al., 2003). The colonies that we recovered from transfection of *rev1* cells did not show loss of the G4 sequence. This suggests that other mechanisms are able to compensate for the loss of REV1 but that in *rev1* cells these are not able to efficiently counteract loss of the plasmid.

Using this assay, we were able to dissect the contribution of the different domains of REV1 to the replication of the G4 containing plasmid. REV1 has two principal activities. It is a deoxycytidyl transferase (Nelson et al., 1996) and also has a noncatalytic function in which its C terminus plays a crucial role in the coordination of TLS by other polymerases (Guo et al., 2003; Ross et al., 2005). Full-length human REV1 complemented the G4 replication defect completely, as it does other *rev1* phenotypes (Edmunds et al., 2008; Ross et al., 2005) (Figure 4E). A DT40 line harboring a REV1 BRCT domain deletion (Figure S2) also exhibited no defect. However, consistent with our observations on replication fork progression (Edmunds et al., 2008), REV1 lacking its C-terminal 100 amino acids did not complement, implicating the polymerase-interacting region in the replication of G4 DNA. Interestingly, a catalytically dead mutant complemented only to about 50% of full-length REV1, suggesting that the catalytic activity also plays a role in G4 DNA replication, perhaps by the incorporation of a nontemplated C opposite G, leading to disruption of the G4 structure (Figure S3).

### Loss of Transcriptional Repression in *rev1* Cells Is Associated with G4-Forming DNA

In the light of the requirement for REV1 in replication of this specific G4 sequence, we speculated that the phenotype observed at the ρ-globin gene could represent a more general loss of repression in the vicinity of sequences with the potential to form G4 structures. In order to test this, we used a microarray to identify targets significantly upregulated in *rev1* cells relative to the WT. Having validated the upregulation of four transcripts in two further independent *rev1* lines by qPCR (Figure S4), we selected genes from the array that were expressed at a low level in WT cells, but that were increased more than 1.4-fold in *rev1* with a t test p value of <0.075, and examined the sequence 1500 bp either side of the annotated transcriptional start site for the presence of G4-forming sequences, using the Quadfinder server (Scaria et al., 2006). As a control, we analyzed a set of genes with similarly low levels of expression in WT but that were not upregulated in *rev1* cells. At least one G4 sequence within the 3 kb window was found in 38% of the control set (Table S1), a similar figure to that obtained in previous analyses of G4 sequences near promoters in the chicken (Du et al., 2007) and human genomes (Eddy and Maizels, 2008; Huppert and Balasubramanian, 2007). Contrastingly, 71% of the targets upregulated in *rev1* cells contained at least one predicted G4-forming sequence in the 3 kb window. Therefore, there is a statistically significant (p < 0.001) association between G4 sequences in the vicinity of the promoter and increased gene expression in *rev1* cells.

### Introduction of the 29 bp ρ-Globin G4 DNA into a Silent Locus Confers Susceptibility to Derepression in *rev1* Cells

**Figure 5:**
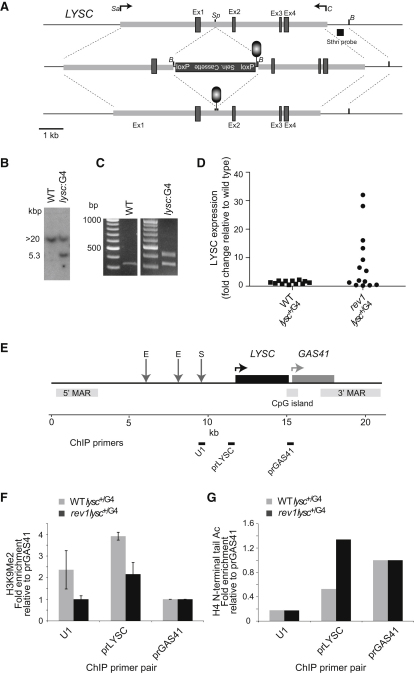
Derepression of Silenced Loci in *rev1* Cells Is Associated with G4 DNA (A) Introduction of the ρ-globin G4 DNA into the *LYSC* locus. The genomic locus was amplified as a SalI (Sa)-ClaI (C) fragment with primers LYSCSalF and LYSCClaR. A linker DNA (G4TplantSph1) containing a BamHI (B) site and the G4 sequence was introduced into the SphI (Sp) site in the first intron of the LYSC gene, so as to be on the feature strand. Correct insertion of the G4 DNA was confirmed by sequencing with primer LYSCG4seq. A bidirectional origin has been demonstrated in the CpG island at the 3′ end of the gene (Phi-van and Strätling, 1999) meaning that the introduced G4 structure will form on the leading-strand template. A puromycin-resistance selection cassette was inserted into the BamHI site. This was then removed by transient expression of Cre recombinase. (B) Southern blot of BamHI-digested DNA showing targeting of one allele of LYSC producing at ∼5 kb band. (C) Confirmation of the presence of the G4 sequence by PCR using primers (LYSCG4F and R) annealing either side of the expected insertion of the G4 DNA (plus the remnants of the loxP recombination sites). (D) qRT-PCR for LYSC expression in clones of WT and *rev1* harboring the ρ-globin G4 DNA in the LYSC locus expressed as the fold change relative to unmanipulated WT DT40. (E) Map of the chicken *LYSC* and *GAS41* loci. E, transcription enhancer element; S, transcription suppressor element; MAR, matrix attachment region (adapted from Myers et al., 2003). The positions of the three pairs of ChIP primers are indicated. (F) H3K9 dimethylation (H3K9me2) at the *LYSC* locus normalized to that at the constitutively active *GAS41* promoter in WT and *rev1* cells harboring the ρ-globin G4 DNA in one allele of the *LYSC* locus (*lysc*^+/G4^). Error bars represent standard error of the mean. (G) H4 N terminal acetylation at the *LYSC* locus in WT and *rev1 lysc*^+/G4^ cells, normalized to the *GAS41* promoter.

In order to test our hypothesis more directly, we conducted an experiment to transplant the G4 sequence from the ρ-globin locus into the developmentally regulated lysozyme C gene. *LYSC*, another well-studied locus (Myers et al., 2006) that is silent in chicken lymphocytes, lacks any endogenous G4 sequence and is unaffected by loss of REV1 in DT40. Like the ρ-globin gene, it has a strong origin of replication at its 3′ end (Phi-van and Strätling, 1999). Using homologous recombination, we inserted the G4-forming sequence and a puromycin resistance selection cassette into the first intron of *LYSC* approximately 500 bp from the transcriptional start site, a position equivalent to that seen in the ρ-globin locus (Figures 5A–5C). Having screened for successful integrants in both WT and *rev1* backgrounds, we removed the selection cassette by Cre-loxP recombination and cultured the clones for 4 weeks. We detected a marked increase in expression of lysozyme in 7 of 14 clones of *rev1* cells carrying the same G4 DNA integration (Figure 5D). Such an effect was not observed in any of 12 WT clones harboring the same integration of the ρ-globin G4 DNA in the *LYSC* locus (Figure 5D). To examine whether this increase in expression was correlated with the epigenetic changes predicted by our model, we examined the H3K9 dimethylation and H4 N-terminal tail acetylation at the *LYSC* promoter in a *rev1 lysc*^+/G4^ clone exhibiting a >30-fold increase in lysozyme messenger RNA (mRNA). Compared to a WT *lysc*^+/G4^, the level of H3K9me2 is decreased, while the level of H4 N-terminal acetylation is increased. Thus, the ρ-globin G4 sequence can trigger loss of repression when inserted into a silenced locus in *rev1*, but not WT, cells.

## Discussion

**Figure 6:**
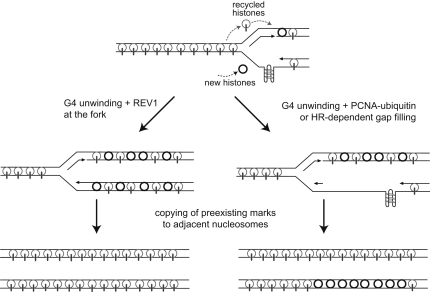
A Model for Loss of Repressive Histone Marks at Sites with G4-Forming Potential in *rev1* Cells Replication is depicted arresting at a G4 DNA on the leading-strand template. Parental histones are shown as light-gray circles, with repressive epigenetic marks represented as gray bars. New histones are shown in black. If REV1 is present, the fork can replicate through the G4 DNA, maintaining processive DNA synthesis and histone deposition. It is not clear whether the presence of REV1 prevents the formation of the structure or assists in its unwinding (see also Figure S2). In the absence of REV1 the fork remains arrested at the G4 DNA, resulting in a postreplicative gap. The DNA synthesis associated with the resolution of this gap and of the G4 DNA is accompanied by new histone incorporation resulting in a tract of chromatin lacking the parental epigenetic marks.

In this work, we demonstrate a link between two important facets of chromosomal replication, the replication of structured DNA and the faithful maintenance of a repressive chromatin environment. Our observations suggest a model (Figure 6) in which the absence of REV1 leads to uncoupling of histone recycling from DNA synthesis at sites capable of forming G4 structures. In turn, this results in the repeated loading of newly synthesized histones that ultimately leads to a permanent loss of repressive epigenetic marks.

### The role of REV1 in Replication of G4 DNA

Sequences capable of forming G4 DNA are abundant throughout the vertebrate genome but are highly enriched at telomeres, the immunoglobulin gene switch regions, and the vicinity of the transcription start site of genes. There is also increasing evidence for the formation of such structures in vivo (reviewed in Lipps and Rhodes, 2009; Maizels, 2006). G4 DNA can block replicative DNA polymerases in vitro (Woodford et al., 1994), and there is now good evidence, particularly from the study of telomeres, that they form and can slow or block replication in vivo (Schaffitzel et al., 2001; Sfeir et al., 2009). The exact correlation between the replication slow zone in the ρ-globin second intron identified by Prioleau et al. (2003) and a robust G4-forming sequence provides further evidence that these sequences pose a challenge to the replicative machinery, even in normal cells.

TLS has been previously implicated in the replication of G/C tracts in both *C. elegans* and human cell lines. Deletion of either Polκ or Polη in a *dog-1* worm (deficient in the FANCJ helicase) resulted in an increased frequency of small deletions in G/C tracts (Youds et al., 2006). More recently, RNA interference (RNAi)-mediated knockdown of Pols η, ι, and κ has been shown to sensitize cells to the G4-stabilizing compound telomestatin and to result in elevated DNA damage associated with the human c-MYC promoter, which contains a G-rich sequence capable of forming G4 structures (Bétous et al., 2009). However, the precise role of TLS in replication of structures that actually contain no damaged DNA remains unclear. In particular, it remains to be shown whether REV1 collaborates with the helicases that have been demonstrated to unwind G4 DNA, such as FANCJ, BLM, and WRN (Fry and Loeb, 1999; London et al., 2008; Sun et al., 1998; Wu et al., 2008), and whether similar epigenetic instability at G4 DNA is triggered by loss of these helicases. It is noteworthy that cells lacking either BLM or WRN exhibit altered expression of genes harboring sequences with G4-forming potential (Johnson et al., 2010), although this effect was mechanistically ascribed to regulation of transcription.

A potential clue to the role of REV1 may come from our observation that not only the C-terminal polymerase-binding region, but also the catalytic activity of the enzyme is required for fully effective replication of the ρ-globin G4 DNA. REV1 is a deoxycytidyl transferase (Nelson et al., 1996) but also a template G-dependent DNA polymerase (Haracska et al., 2002). This suggests a possible model (Figure S3) in which the ability of REV1 to produce a tract of dC bases with minimal reference to the template may destabilize the G4 structure through base pairing between newly synthesized dC and template dG. The C terminus of the protein could then coordinate the handoff to other TLS polymerases, allowing extension of this dC-rich primer and replication of the G4 sequence.

### Replication Impediments and Epigenetic Stability

Recycling of parental histones is likely to play a key role in the propagation of epigenetic memory but requires tight coupling between histone displacement and redeposition in order that the register between histone marks and underlying DNA sequence is not lost. Such coupling is likely to be mediated by histone chaperones, notably Asf1 (reviewed in Annunziato, 2005; Groth et al., 2007; Margueron and Reinberg, 2010). Indeed, very recent evidence suggests that Asf1 can buffer histones displaced by hydroxyurea-induced replication arrest, leading to the suggestion that replication stress may jeopardize proper chromatin restoration and thereby trigger epigenetic changes in daughter cells (Jasencakova et al., 2010). Here, we provide evidence that replication impediments can indeed lead to epigenetic change, although in our model it is failure to use recycled histones during gap filling rather than unscheduled deposition that underlies a loss of epigenetic information.

Our computer model predicts that histone mark propagation is likely to be sufficiently robust to deal with such gaps occurring sporadically, as would be caused by DNA damage. At levels of damage compatible with cell survival, the model suggests that spreading of histone methylation back into the demethylated gap would result in “healing” of the repressive chromatin environment. However, for G4 DNA, it seems likely that the repeated deposition of newly synthesized histones swamps this ability to restore the pre-existing chromatin environment and ultimately leads to derepression. It is also conceivable that spreading of histone demethylation can occur if the tract of demethylated histones is sufficiently long. It seems likely that this spreading would be limited by chromatin domain insulators, such as those marked by HSA and HS4 (Figure 1A), resulting in switching of the histone methylation state of the whole domain. Such behavior has been proposed on theoretical grounds (Dodd et al., 2007) and may explain how only two identifiable G4 DNA sequences in the condensed chromatin region can nonetheless lead to loss of H3K9 dimethylation across the whole 15 kb domain.

It is noteworthy that derepression in the absence of REV1 is not seen in all silenced loci. Notably, the expression of *HOX* genes, known to be under the control of the polycomb repressive complexes, does not appear to be affected by loss of REV1 (data not shown). This may reflect different mechanistic approaches to the generation and maintenance of particular forms of silencing, which may include specific DNA signals, the use of RNA interference, and histone recycling. Conversely, the heterochromatin region in the β-globin locus does appear to be affected, even though it has recently been proposed that RNAi plays a role in repression of this sequence (Giles et al., 2010). It will therefore be interesting to explore the relationship between histone recycling and mechanisms such as RNAi in the initiation and maintenance of gene repression, possibly using REV1 deficiency as a tool.

Finally, dysregulation of gene expression is common in many cancers. Our observations suggest one possible mechanism by which a single mutation in a pathway promoting genetic stability could lead to more widespread epigenetic instability.

## Experimental Procedures

### DT40 Strains, Culture, and Transfection

DT40 cells were propagated and transfected as previously described (Simpson and Sale, 2003). DT40 mutants used in this work have also been described previously (Arakawa et al., 2006; Edmunds et al., 2008; Kawamoto et al., 2005; Ross et al., 2005; Simpson and Sale, 2003; Takata et al., 2001).

### Chromatin Immunoprecipitation

Chromatin immunoprecipitation (ChIP) was performed as described (Aparicio et al., 2005) using formaldehyde (FA) crosslinking to trap protein-DNA complexes, with minor modifications detailed in the Supplemental Experimental Procedures. PCR primers for ChIP qPCR are listed in Table S2.

### Antibodies for ChIP

The following antibodies were used: anti-H3K9me2, Millipore ChIPAb+ catalog number 17-648; anti-H3K9/K14ac, Millipore ChIPAb+ catalog number 17-615; anti-H3K4me3, Cell Signaling Technology catalog number 9727; anti-H3, Cell Signaling Technology catalog number 2650; and anti-acetylH4, Millipore ChIPAb+ catalog number 17-630. This polyclonal antibody recognizes acetylation of H4K5, 8, 12, and 16 and has been previously used (as Upstate catalog number 06-598) to monitor acetylation of H4 during histone deposition (Lande-Diner et al., 2009). A negative control for ChIP was provided by normal rabbit IgG (Millipore).

### ChIP qPCR and Data Analysis

Quantitative PCR was performed in real time with SYBR green. ChIP DNA (2.5 μl) was used in each reaction, with 400 nM primer mix and 12.5 μl 2xSYBR-green qPCR ready-mix (Invitrogen). The reaction was carried out on a ABI Prism real-time cycler with the following program: 50°C for 2 min, 90°C for 10 min, and 40 cycles of 90°C for 15 s (denaturation), 60°C for 1 min (annealing and extension). Each reaction was performed in duplicate. ChIP results were normalized to the positive control anti-H3 antibody with the formula 2^–(Ct(Ab)-Ct(H3))^. In control experiments to initially validate the protocol, immunoprecipitation with the normal rabbit IgG antibody recovered extremely low amounts of material (less than 0.1% of the H3 signal and less than 0.05% of input). Data from the β-globin locus was further normalized to the hypersensitive site (HS4) of the β-globin locus, which has been previously shown to contain high levels of H4 and H3 N-terminal acetylation (Litt et al., 2001b). This was found to allow reproducible comparison between different extracts. For the *LYSC* locus, normalization of the specific ChIP signal was to total H3, then to the promoter of the adjacent, constitutively active *GAS41* locus. Absolute enrichment relative to total H3 of H3K9me2 and H4 N-terminal acetylation at the *GAS41* promoter was found to be similar in WT and *rev1* cells.

### qRT-PCR

RNA was extracted with Trizol (Invitrogen) according to the manufacturers' instructions. cDNA was prepared with 5 μg mRNA with Super RT (HT Biotechnology, Cambridge, UK) and oligodT primer in a final volume of 40 μl. qPCR reactions were performed as described above, with 2.5 μl cDNA per reaction. Quantitation was relative to β-actin (cDNA diluted 1/100) with the exception of LYSC, which was relative to the adjacent GAS41 gene (see Table S2 for primer sequences). The efficiency of amplification was verified to be close to 1 (i.e., a Ct change of 1 reported a 2-fold change in concentration of template) for the control primers with a standard curve of cDNA dilutions.

### DNA Methylation Analysis

Five micrograms of genomic DNA, quantified by nanodrop spectrophotometer, was cut with 5 units HpaII, BamHI, or MspI for 6 hr at 37°C. After phenol/chloroform extraction, DNA was precipitated overnight at –20°C with sodium acetate and ethanol before analysis with qPCR. HpaII and MspI are isoschizomers, but HpaII is blocked by CpG methylation whereas MspI is not; therefore, we could verify the assay by amplifying from the hypersensitive site showing no HpaII enrichment relative to MspI. Data from the ρ-globin promoter was normalized to BamHI digested DNA to control for differences in genomic DNA preparations (there are no BamHI sites within the expected amplicon).

### Circular Dichroism Spectroscopy

Oligonucleotides corresponding to the full-length ρ-globin G4 and the shorter sequence lacking the first run of Gs (Figure 4A) were synthesized and purified by desaltion (Sigma), resuspended in TE buffer, and diluted to a final concentration of 10 μM before heating to 95°C for 5 min to denature secondary structure. At this point, either 2M KCl was added to a final concentration of 100 mM or an equivalent volume of nuclease-free H_2_O was added. The oligos were then left to cool overnight at room temperature. Spectroscopy was performed on a Jasco J-810 spectrometer at room temperature with a bandwidth of 2 nm, a response of 1 s, a data pitch of 0.2 nm and a scanning speed of 50 nm/min. Scans were performed over the range 200 to 320 nm. Curves for each oligo were processed by subtracting the trace produced by TE buffer (with or without KCl) and smoothing with the software provided by the manufacturer.

### Replicating Plasmid Assay

For creation of the G4-containing replicating plasmid, oligonucleotides coding the ρ-globin G4 sequence (RGG4LeadF and RGG4LeadR) were ligated into pQ1 (Szüts et al., 2008) as an EcoRI fragment. Sequencing was used to select plasmids where the G4 DNA was on the leading-strand template relative to the Gal origin (Figure 5). A second plasmid placing the G4 on the lagging-strand template was made with oligonucleotides with EcoRI and PstI cohesive ends (RGG4LagF and RGG4LagR). Oligonucleotide sequences are given in Table S2. The plasmid replication assay was performed as described (Szüts et al., 2008) with minor modifications: 1 μg control G4-free pQ2 plasmid (conferring kanamycin resistance) was used per transfection along with 1 μg G4-containing plasmid, (conferring ampicillin resistance); DpnI-digested plasmid was used to transform Invitrogen E-shot electrocompetent cells (catalog number 18290-015); and an equal amount of cells was plated out onto kanamycin and ampicillin plates.

### Computer Modeling

The computer simulation “Zippee” is a Java applet that will run on most web browsers with the latest implementations of Java. (We have tested it on Internet Explorer 7, Firefox 3, and Safari 4). It can be found at http://www.cl.cam.ac.uk/∼calr3/zippee/. The Java code is available on request. A detailed description of the algorithm can be found in Supplemental Information.

### Microarray

Microarray analysis was performed on three independent *rev1* and WT lines simultaneously. RNA was extracted with Trizol (Invitrogen). The cDNA labeling and microarray hybridization was carried out by the Cambridge University School of Clinical Medicine Department of Metabolic Science with the Affymetrix Chicken Genome array. For analysis of the data, targets showing increased mean probe intensity in *rev1* relative to the WT were sorted according to t value. Targets were selected whose initial mean probe intensity value was below 7 and whose upregulation was at least 40% and significant at p < 0.075. We sorted the list by decreasing change in expression relative to WT and downloaded 1500 kb either side of the transcriptional start site as annotated in Ensembl (as at 31.3.2010), discarding targets that either had significant unsequenced regions or could not be found in the Ensembl database. For our control set, we took the targets with the highest t value (i.e., no change between WT and *rev1*) and selected for those that were expressed at a level below a mean WT probe intensity level of 7. From this list, targets were selected at random and analyzed in the same way as the upregulated set. The sequence data from both sets was then analyzed with the Quadfinder server (http://miracle.igib.res.in/quadfinder/) (Scaria et al., 2006) to search for potential G4 forming motifs on either strand according to the consensus G_(3-5)_L_(1-7)_G_(3-5)_L_(1-7)_G_(3-5)_L_(1-7)_G_(3-5)_. We used Fisher's Exact Test to determine whether there was a significant difference between the numbers of G4 motifs found in the upregulated and the control sets.

## Supplemental Information

Document S1. Supplemental Experimental Procedures, Four Figures, and One Table

Table S1. Correlation of Increased Gene Expression in rev1 Cells with the Presence of Sequences Capable of Forming G4 DNA in a Region ± 1500 bp of the Promoter of the Gene, Related to the Results
