# Supplementary material for: Epigenetic Instability due to Defective Replication of Structured DNA
Source: Mol Cell. 2010 Dec 10;40(5):703–13. doi: 10.1016/j.molcel.2010.11.009 (PMC3145961; doi:10.1016/j.molcel.2010.11.009)
Supplement: Document S1. Supplemental Experimental Procedures, Four Figures, and One Table [file mmc1.pdf]

Molecular Cell, ***Volume 40***

Supplemental Information

Epigenetic Instability due to Defective Replication of Structured DNA

Peter Sarkies, Charlie Reams, Laura J. Simpson, and Julian E. Sale

SUPPLEMENTAL EXPERIMENTAL PROCEDURES

Chromatin immunoprecipitation

Modifications to the ChIP procedure of Aparicio et al. (Aparicio et al., 2005). Lysates were prepared and the DNA was fragmented by sonicating to a size of <1.5 kb with an average of 400-600 bp as shown by analysis on a 1.5 % agarose gel. An amount of cross-linked lysate calculated to contain approximately 25-50 µg of DNA was diluted to a total volume of 500 µl with FA lysis buffer (Aparicio et al., 2005) before incubation overnight at 4 ˚C with the ChIP antibodies (see below). The antibody complexes were then precipitated by incubation with 30 µl Protein G magnetic beads (New England Biolabs) for 2 hours at 4˚C. After sequential washing of the beads with FA lysis buffer, FA lysis buffer with 0.5M NaCl, a LiCl-based chromatin immunoprecipitation wash buffer (Aparicio et al., 2005) and TE buffer, complexes were eluted by shaking in an Eppendorf Thermomixer at 65 ˚C for 30 minutes in 100 µl ChIP elution buffer (1 % SDS, 100 mM $NaCO_3$). Following this, the mixture was treated with 5 µg Proteinase K (1 mg/ml solution, Sigma), for 2 hours at 42 ˚C and cross-links were reversed by incubating for 4 hours at 65 ˚C. Finally, DNA was precipitated using sodium acetate and ethanol and analysed by quantitative PCR using primers listed in Table S2.

Computer simulation of histone recycling

The simulation uses as its biological basis a similar model to that used by Thon and colleagues (Dodd et al., 2007), but employs stochastic simulation rather than one based on differential equations. For simplicity, only three states of nucleosomes are considered, modified, unmodified and naïve, the latter being the state of a newly synthesised histone. The simulation uses a uniform tract of stably modified histones as its template and histone replication is semiconservative. Thus, for every two nucleosomes deposited on the newly synthesised DNA, one is from the parental duplex and one is newly synthesised. However, the order in which they are arranged is random, reflecting the stochastic nature of histone segregation onto the daughter strands. Once this is complete, the modified histones' marks are copied onto adjacent naïve nucleosomes. Additionally there is a finite probability that the modified mark can spread more than one nucleosome away (two-place copying), thus endowing the system with robustness in the face of errors. The model can 'stall' replication with a defined per nucleosome probability and, to test our hypothesis for histone deposition during the restoration of post-replicative gaps, it deposits a tract of naïve histones in the gap, the length of the gap being another variable parameter. The implementation is divided into three stages:

Stage 1- Replication

Nucleosomes are removed from the template. For every two successive nucleosomes removed, one retains its original mark, and the other assumes an unmodified 'naïve' state. This process is subject to random error limited to one nucleosome, given by the parameter '*probability of mark misincorporation*'. If a stall occurs, however, with probability determined by the parameters '*probability of roaming stall*' or '*probability of fixed stall*', the simulation inserts an integral number of naïve histones with the parameters '*gap length*' and '*gap length variance*' specifying Gaussian mean and variance respectively.

Stage 2 - Mark copying.

The final state of each naïve nucleosome is determined by the states of the adjacent marked nucleosomes. 'Stop' and 'go' marks, corresponding to repressed and active nucleosome states respectively, can each copy onto the naïve nucleosomes with probability determined by the parameters '*probability of stop copying*' and '*probability of go copying*'. Additionally, the parameter *rho* determines the probability of a mark copying to a naïve nucleosome two places away: *rho* is the ratio of probability of one place copying to probability of two place copying.

Stage 3 - Completion

The nucleosomes are returned to the template. At this point, any nucleosome that has not obtained a modification, i.e. remains naïve, will acquire the modification 'go', to reflect persistence of H4 N terminal acetylation. The simulation is now ready for another round of division.

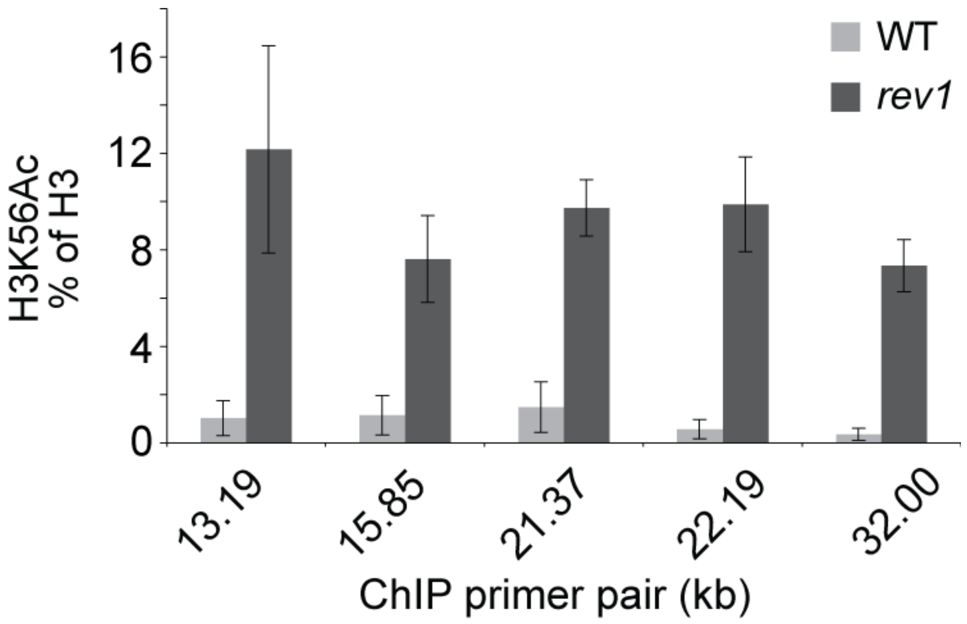

Figure S1. Increased Acetylation of H3K56 in the β-Globin Locus of *rev1* Cells, Related to Figure 1. The location of the ChIP primers is given in Figure 1A and results are normalised to total H3, but not then normalised again to the hypersensitive site as no information exists on the global pattern of H3K56ac in vertebrates. Error bars represent standard error of the mean.

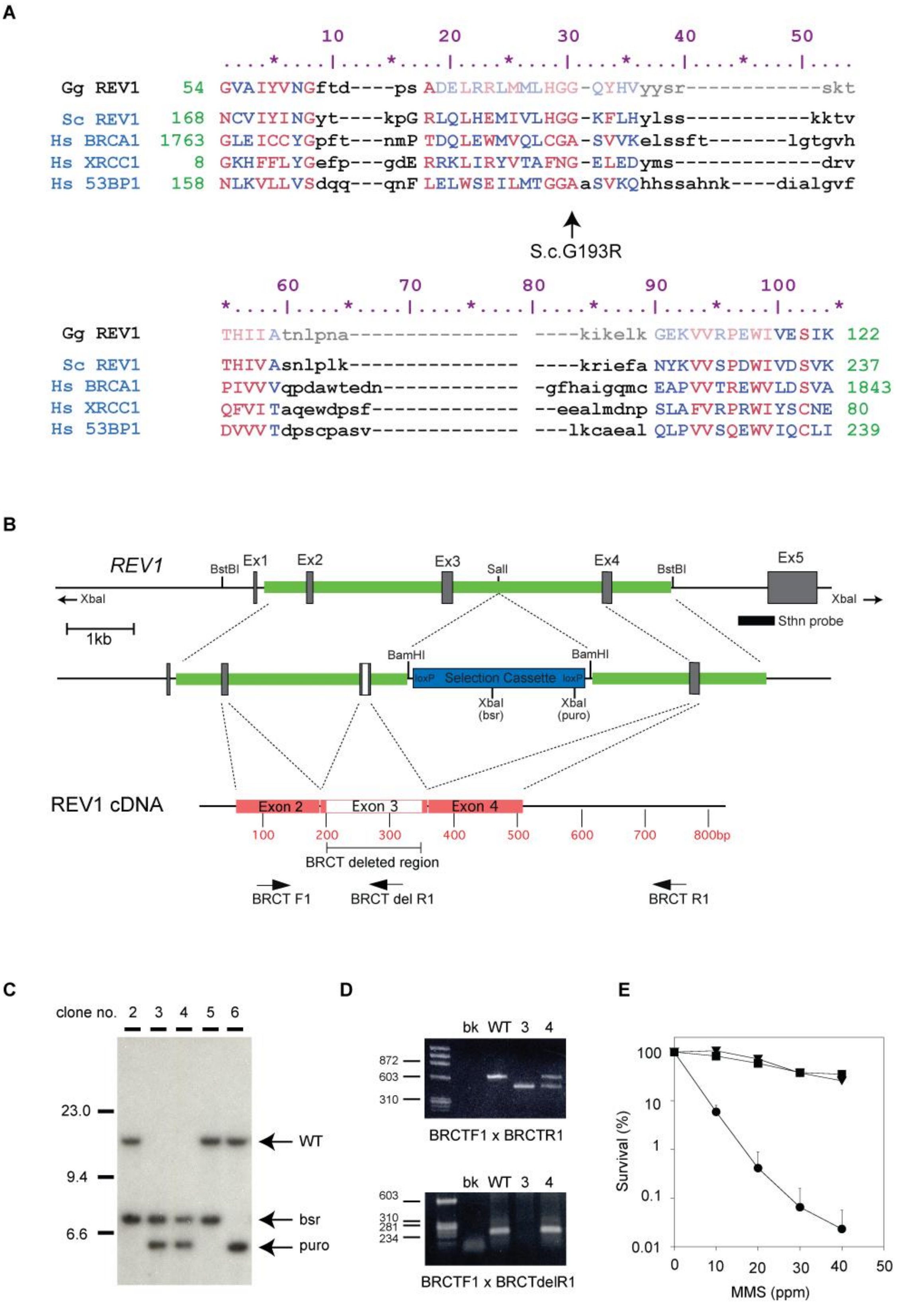
A
10 20 30 40 50
Gg REV1 54 GVAIYVNGftd---ps ADELRRLMMLHGG-QYHVyysr------------skt
Sc REV1 168 NCVIYINGyt----kpG RLQLHEMIVLHGG-KFLHylss-----------kktv
Hs BRCA1 1763 GLEICCYGpft---nmP TDQLEWMVQLCGA-SVVKelssft-------lgtgvh
Hs XRCC1 8 GKHFFLYGefp---gdE RRKLIRYVTAFNG-ELEDyms-------------drv
Hs 53BP1 158 NLKVLLVSdqq---qnF LELWSEILMTGGAaSVKQhhssahnk----dialgvf
S.c.G193R
60 70 80 90 100
Gg REV1 THIIAtnlpna-------------- ----kikelk GEKVVRPEWIVESIK 122
Sc REV1 THIVAsnlplk-------------- ----kriefa NYKVVSPDWIVDSVK 237
Hs BRCA1 PIVVVqpdawtedn----------- -gfhaigqmc EAPVVTREWVLDSVA 1843
Hs XRCC1 QFVITaqewdpsf------------ --eealmdnp SLAFVRPRWIYSCNE 80
Hs 53BP1 DVVVTdpscpasv------------ ---lkcaeal QLPVVSQEWVIQCLI 239
B
REV1
BstBI Ex1 Ex2 Ex3 SalI Ex4 BstBI Ex5
XbaI
XbaI
1kb
Sthn probe
BamHI
BamHI
loxP Selection Cassette loxP
XbaI (bsr)
XbaI (puro)
REV1 cDNA
Exon 2 Exon 3 Exon 4
100 200 300 400 500 600 700 800bp
BRCT deleted region
BRCT F1
BRCT del R1
BRCT R1
C
clone no. 2 3 4 5 6
23.0
9.4
6.6
WT
bsr
puro
D
bk WT 3 4
872
603
310
BRCTF1 x BRCTR1
bk WT 3 4
603
310
281
234
BRCTF1 x BRCTdelR1
E
100
10
1
0.1
0.01
Survival (%)
0 10 20 30 40 50
MMS (ppm)

Figure S2. ***In Situ*** **Disruption of the BRCT Domain of REV1 in DT40, Related to Figure 4.** We have previously reported that reconstitution of *rev1* DT40 cells with a construct expressing human REV1 lacking the N terminal 332 amino acids was able to fully complement phenotypes of the mutant including damage sensitivity and defective replication fork progression after DNA damage, suggesting that the BRCT domain is dispensable for DNA damage tolerance (Edmunds et al., 2008; Ross et al., 2005). However, we suggested that the discrepancy between these observations and the apparently essential role of the BRCT domain in yeast (Nelson et al., 2000) might be explained by our ectopic expression bypassing the requirement for the BRCT domain (Ross et al., 2005). To address this we created a DT40 *rev1* BRCT mutant in which the protein is expressed under its natural promoter.

A. Alignment of the chicken (Gg) REV1 BRCT domain with the BRCT domains of yeast (Sc) REV1 and human (Hs) BRCA1, XRCC1 and 53BP1 made with CDD (Marchler-Bauer et al., 2009). The position of G193, mutated to R in the *Saccharomyces cerevisiae rev1-1* allele is indicated. The amino acids deleted in our REV1 BRCT mutant are shown in light type. B. Schematic for gene targeting. A 6.059 kb genomic *Bst*BI fragment containing exons 2 – 4 was amplified with primers BRCT5F1 and RBRCT3R2. A 792 bp *Spe*I fragment containing exon 3 was removed. It was replaced with an modified *Spe*I fragment in which amino acids 69 – 116 of exon 3 had been removed . This fragment was generated by amplifying from the 5' *Spe*I site to amino 68 (2BRCTF1 and 2BRCTR1) and from amino acid 117 to the 3' *Spe*I site (2BRCTF2 and 2BRCTR2). The two products were joined by an *Apa*I site. A *Bam*HI linker was cloned in the *Sal*I site in the intron between exons 3 and 4 to allow introduction of the antibiotic selection cassette. The resulting cDNA is shown in pink with the position of the PCR primers used in panel D indicated. C. Southern blot showing second allele targeting. The first allele was targeted with a construct containing a blasticidin resistance cassette (bsr). The second construct contained a puromycin-resistance cassette (puro). Clones 2 and 5 have not targeted in the second round, while clone 6 has undergone retargeting of the initially targeted allele. Clones 3 and 4 have undergone targeting of both alleles. D. RT-PCR to confirm disruption of exon 3. Primer pairs are indicated on the cDNA map in panel B and detailed, along with the other primers used, in Supp. Table 2. The crossover in clone 4 has resulted in integration of the puro resistance cassette but no alteration of exon 3. Clone 3 is correct. The selection cassettes were removed by transient expression of a plasmid expressing Cre recombinase, which targets the indicated loxP sites. E. Colony survival following exposure to methyl methane sulphonate (MMS). Wild type = boxes, *rev1* = circles and *rev1*[ΔBRCT] clone #3 = inverted triangles.

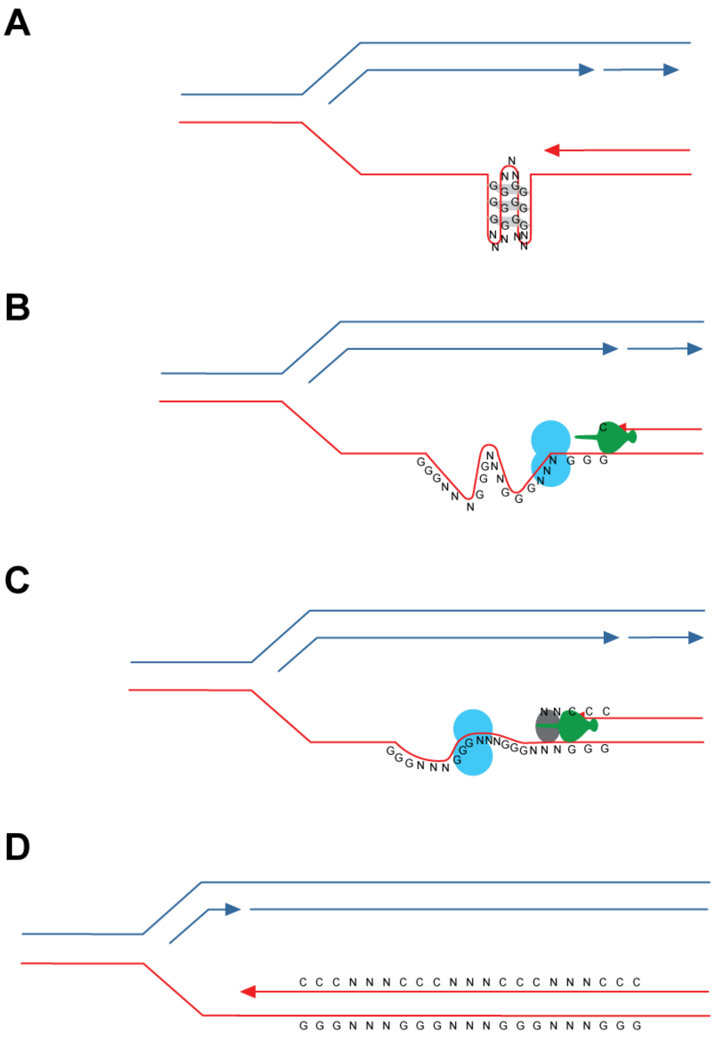

Figure S3. A Model for the Role Played by REV1 in the Replication of G4 DNA, Related to Figure 4 and the Discussion. A. Replication stalled at a G4 structure that has formed on the leading strand template between the replicative helicase and polymerase. B. A specialised helicase (blue circles) starts to unwind the G4 exposing the first run of Gs. The deoxycytidyl transferase activity of REV1 may facilitate stabilisation of these exposed dG bases by extending the primer terminus with dC. This prevents the G4 refolding since three G runs does not support G quadruplex formation (Fig. 2B). The polymerase-interaction domain in the extreme C terminus of REV1 then coordinates the completion of the replication of the G4 DNA.

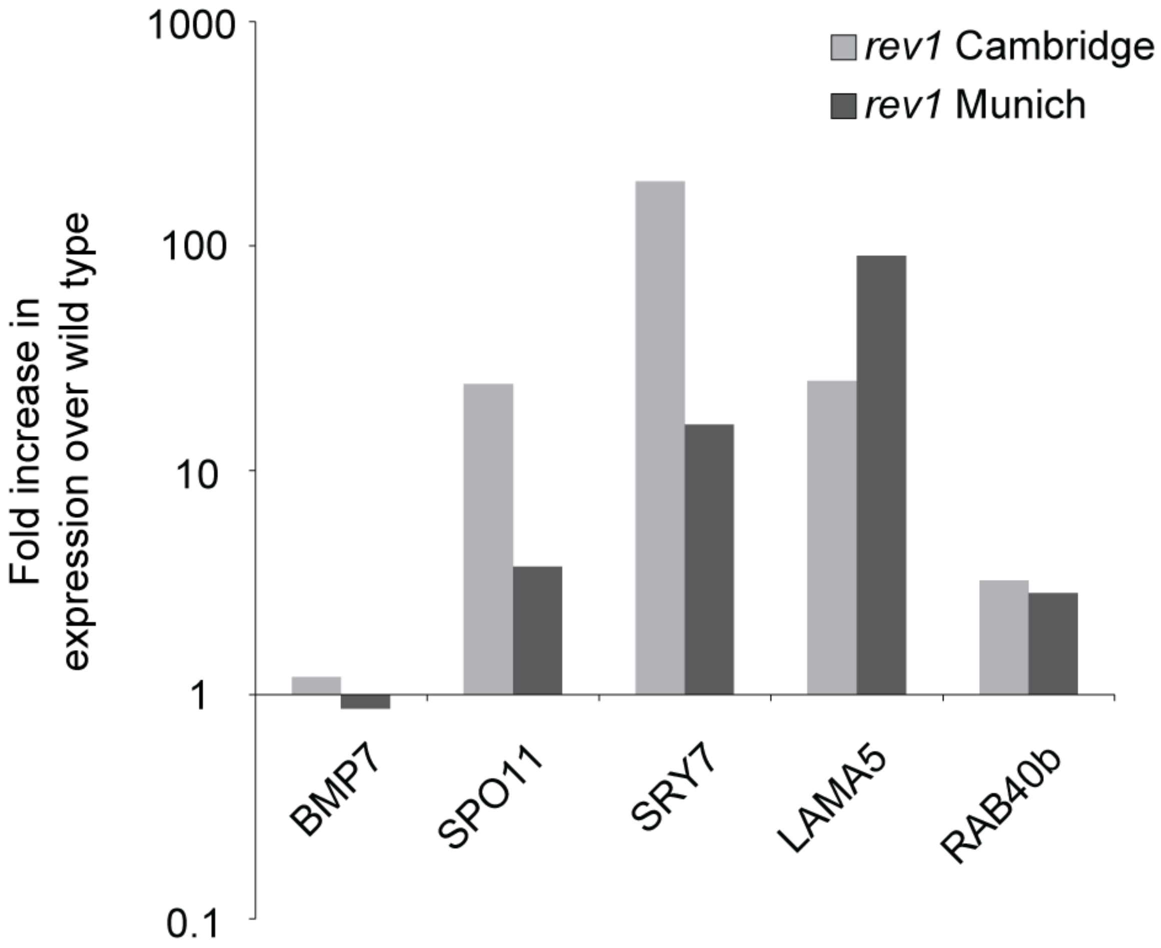

Figure S4. Validation by qPCR of Selected Genes Identified to be Upregulated in *rev1* Cells in the Microarray Analysis, Related to the Results. qPCR of the indicated genes performed in two clones of *rev1* cells not used in the microarray experiment. BMP7 was not upregulated in *rev1* cells in the array and is included as a control. Primers used are listed in Table S2.

Table S1. Correlation of Increased Gene Expression in *rev1* Cells with the Presence of Sequences Capable of Forming G4 DNA in a Region ± 1500 b.p. of the Promoter of the Gene, Related to the Results. Table S1 is an Excel workbook with two sheets – one for the identified induced genes and the second for the control set. mRNA from three clones of wild type and *rev1* were analysed. The raw expression data is given in columns E to J. The position of potential G4 sequences is given in column R relative to the transcriptional start site. nd = not done; this was because either the probe was not annotated, had multiple hits in the genome or the available genomic sequence had gaps. The sequence of the potential G4 is given 5' – 3' in column S and the strand, either feature (F) or template (T) in column T. The 'Induced set' sheet lists all the genes whose basal expression in wild type is < 7 log units and which are > 1.4-fold higher in *rev1* cells. Column U presents the analysis of the number of G4 DNA-containing sequences for all the genes meeting these criteria. Column V only includes those with a t-test p value of < 0.075. The control set of genes were selected at random from a pool of genes expressed at < 7 log units in wild type cells and whose expression was unchanged in the absence of REV1. The figure of 38% of these genes containing a potential G4 DNA is very similar to the figure obtained in previous analyses of G4 sequences near promoters in the chicken (Du et al., 2007) and human genomes (Eddy and Maizels, 2008; Huppert and Balasubramanian, 2007).

Table S2. Related to the Experimental Procedures

Oligonucleotides

ChIP and qPCR primers

Name	Sequence	Reference
8.99F	GGGCCCAATGAACCAGAAA	(Litt et al., 2001)
8.99R	TGTTCCCCAGCAACGCA	(Litt et al., 2001)
10.35F	GGAACAAGTTGGCAAGGTCCTAT	(Litt et al., 2001)
10.35R	TCTTCTGCCCTGCCCGTAT	(Litt et al., 2001)
13.19F	GAATGTGTCCATCTGCCCTCAT	(Litt et al., 2001)
13.19R	GGGAAGCCATCCCTGCA	(Litt et al., 2001)
15.85F	CAGCAGACGCTGTGGTGAA	(Litt et al., 2001)
15.85R	CTTGCAGGATGCAGACTGGA	(Litt et al., 2001)
17.76F	TGTTATCGCACACACACACACTTT	(Litt et al., 2001)
17.76R	GACAGGGATGTTCTTCCTCTGAA	(Litt et al., 2001)
19.37F	TGCTGGCAGTCACACGTCA	(Litt et al., 2001)
19.37R	AACGAAAACACAATAAATAATAGCAATGA	(Litt et al., 2001)
21.37F	CTCTGTGCTCAGCATCCTTCAAT	(Litt et al., 2001)
21.37R	CCTTTCGGCACTTTCTTCCTTT	(Litt et al., 2001)
22.19F	CAGGACAGCATGGACGTGG	(Litt et al., 2001)
22.19R	TTCTGAACGCTGTGACTTGGA	(Litt et al., 2001)
32.00F	TCAGTGTGCACAAGGTGTGG	(Wang et al., 2005)
32.00R	GAAGGGTGAGGGAAGTGCC	(Wang et al., 2005)
prGAS41F	CGTGAACTGCGCGAAGAAG	(Myers et al., 2003)
prGAS41R	CCCCCGCCACCTACCA	(Myers et al., 2003)
prLYSCF	CTGTGGCTTAGCCAATGTGGTA	(Myers et al., 2003)
prLYSCR	AAACGCCCTCTTGAGTATACAGAAA	(Myers et al., 2003)
U1F	AATCTAAGTTTGAAGACAATGAGGTTTTAG	(Myers et al., 2003)
U1R	AGCAAAAAGACATGTAAATACCATAGCT	(Myers et al., 2003)
RhoexpF	GACAATGCAAGGATGGAGTGG	(Wang et al., 2005)
RhoexpR	GAGATGCTCTTTCTGCAGGAAAAT	(Wang et al., 2005)
BactexpF	TGTCCACCTTCCAGCAGATGT	(Myers et al., 2006)
BactexpR	AGTCCGGTTTAGAAGCATTTGC	(Myers et al., 2006)
LysCexpF	GAAGCGTCACGGACTTGATAACTA	(Myers et al., 2006)
LysCexpR	CCCATCGGTGTTACGGTTTG	(Myers et al., 2006)
GAS41expF	TTTCAAGATCCTACTGCCATGATG	(Myers et al., 2006)
GAS41expR	TTCAAGATCTGCAAACTCTGTTTCA	(Myers et al., 2006)
SPO11expF	CTTTTGAGGCTCACCAGCTC	this study
SPO11expR	CACACTGGCTGACAAGCAAT	this study
BMP7expF	ACTGTGAAGGAGAATGCGCT	this study
BMP7expR	GAGCACTGAGATGGCATTGA	this study
SRY9expF	AGGACAAATGCATCTCCGAC	this study
SRY9expR	TTGGGGAAGGTGTTCTCTTG	this study

LAMA5expF	GAAGAGGAACAGCAGCAACC	this study
LAMA5expR	TGCTCAGGGTCTCTTCCACT	this study
RAB40BexpF	CGACTTCCTGCTGAAGTTCC	this study
RAB40BexpR	AGAATCTCCCTTGCCCTGAT	this study

G quadruplex Experiments

RGG4LagF	AATTCGGGGAGTAAAAGGGAGCGGGGTGCTGGGGCTGCA
RGG4LagR	GCCCCAGCACCCCTCTCCCTTTTACTCCCCG
RGG4LeadF	AATTCGGGGAGTAAAAGGGAGCGGGGTGCTGGGGAG
RGG4LeadR	AATTCTCCCCAGCACCCCGCTCCCTTTTACTCCCCG
GQCDG4	AAGGGGAGTAAAAGGGAGCGGGGTGCTGGGGAA
GQCDG3	AGGGAGCGGGGTGCTGGGGA
G4TplantSph1F	CGGGGAGTAAAAGGGAGCGGGGTGCTGGGGCGGATCCGC ATG
G4TplantSph1R	CGGATCCGCCCCAGCACCCCGCTCCCTTTTACTCCCCGC ATG
LYSCSalF	GCGTCGACCAGATGACTATGACTACTGGCAGG
LYSCClaR	GCATCGATACAGCCTTCTCACAGCTGTAGTTC
LYSCG4seq	CATGCCTCAGGATGCTGGCGGGCAGGC
LYSCG4F	GAGAGGGGATCATAGAAACAAGGAG
LYSCG4R	CTTTCTGCATCAGTACCTGCTCAC

REV1 BRCT Domain Mutant

RBRCT5F1	GCACGCGTGAAAGTGAGAGAGATAATATAGCAGC
RBRCT3R2	ACAAAACACATCGTTCGAAGTAAAAGGC
2BRCTF1	GAACATACTAGTTAATCTAATAC
2BRCTR1	TAGGGCCCATCAGCTGAGGGATCTATAA
2BRCTF2	TAGGGCCCATAGTGGAAAGGTAAGTTCAGGC
2BRCTR2	CTGAGAACTAGTCCACTGACTGG
3ProbeF1	GGTAACTAGATGACAAAAATGGC
3ProbeR1	CCAGCTTTGTAAATCCCAACCCG
BRCTF1	GGATCAGTTCCGGTCAGATTCAGC
BRCTR1	GCACCAAGCAATCCTGTGTCTTTAAGG
BRCTdelR1	GGCAGATTTGTGGCAATGATGTGCG

SUPPLEMENTAL REFERENCES

Aparicio, O., Geisberg, J.V., Sekinger, E., Yang, A., Moqtaderi, Z., and Struhl, K. (2005). Chromatin immunoprecipitation for determining the association of proteins with specific genomic sequences in vivo. Curr Protoc Mol Biol *Chapter 21*, Unit 21 23.

Dodd, I.B., Micheelsen, M.A., Sneppen, K., and Thon, G. (2007). Theoretical analysis of epigenetic cell memory by nucleosome modification. Cell *129*, 813-822.

Du, Z., Kong, P., Gao, Y., and Li, N. (2007). Enrichment of G4 DNA motif in transcriptional regulatory region of chicken genome. Biochem Biophys Res Commun *354*, 1067-1070.

Eddy, J., and Maizels, N. (2008). Conserved elements with potential to form polymorphic G-quadruplex structures in the first intron of human genes. Nucleic Acids Res *36*, 1321-1333.

Edmunds, C.E., Simpson, L.J., and Sale, J.E. (2008). PCNA Ubiquitination and REV1 Define Temporally Distinct Mechanisms for Controlling Translesion Synthesis in the Avian Cell Line DT40. Mol Cell *30*, 519-529.

Huppert, J.L., and Balasubramanian, S. (2007). G-quadruplexes in promoters throughout the human genome. Nucleic Acids Res *35*, 406-413.

Litt, M.D., Simpson, M., Recillas-Targa, F., Prioleau, M.N., and Felsenfeld, G. (2001). Transitions in histone acetylation reveal boundaries of three separately regulated neighboring loci. EMBO J *20*, 2224-2235.

Marchler-Bauer, A., Anderson, J.B., Chitsaz, F., Derbyshire, M.K., DeWeese-Scott, C., Fong, J.H., Geer, L.Y., Geer, R.C., Gonzales, N.R., Gwadz, M., *et al.* (2009). CDD: specific functional annotation with the Conserved Domain Database. Nucleic Acids Res *37*, D205-210.

Myers, F.A., Chong, W., Evans, D.R., Thorne, A.W., and Crane-Robinson, C. (2003). Acetylation of histone H2B mirrors that of H4 and H3 at the chicken β-globin locus but not at housekeeping genes. J Biol Chem *278*, 36315-36322.

Myers, F.A., Lefevre, P., Mantouvalou, E., Bruce, K., Lacroix, C., Bonifer, C., Thorne, A.W., and Crane-Robinson, C. (2006). Developmental activation of the lysozyme gene in chicken macrophage cells is linked to core histone acetylation at its enhancer elements. Nucleic Acids Res *34*, 4025-4035.

Nelson, J.R., Gibbs, P.E., Nowicka, A.M., Hinkle, D.C., and Lawrence, C.W. (2000). Evidence for a second function for Saccharomyces cerevisiae Rev1p. Mol Microbiol *37*, 549-554.

Ross, A.L., Simpson, L.J., and Sale, J.E. (2005). Vertebrate DNA damage tolerance requires the C-terminus but not BRCT or transferase domains of REV1. Nucleic Acids Res *33*, 1280-1289.

Wang, J., Liu, H., Lin, C.M., Aladjem, M.I., and Epner, E.M. (2005). Targeted deletion of the chicken β-globin regulatory elements reveals a cooperative gene silencing activity. J Biol Chem *280*, 23340-23348.
